# Interface Synergistic Effect of NiFe-LDH/3D GA Composites on Efficient Electrocatalytic Water Oxidation

**DOI:** 10.3390/nano14201661

**Published:** 2024-10-16

**Authors:** Jiangcheng Zhang, Qiuhan Cao, Xin Yu, Hu Yao, Baolian Su, Xiaohui Guo

**Affiliations:** 1 Key Lab of Synthetic and Natural Functional Molecule Chemistry of Ministry of Education, The College of Chemistry and Materials Science, Northwest University, Xi’an 710069, China; zjc18096314108@126.com (J.Z.); caoqiuhan@126.com (Q.C.); yuxin6123@126.com (X.Y.); 202020797@stumail.nwu.edu.cn (H.Y.); 2 Department of Inorganic Chemistry, University of Namur, 61 rue de Bruxelles, B-5000 Namur, Belgium; bao-lian.su@unamur.be

**Keywords:** NiFe-LDH, graphene aerogel, coupling effect, OER, activity

## Abstract

Currently, NiFe-LDH exhibits an excellent oxygen evolution reaction (OER) due to the interaction of the two metal elements on the layered double hydroxide (LDH) platform. However, such interaction is still insufficient to compensate for its poor electrical conductivity, limited number of active sites and sluggish dynamics. Herein, a feasible two-step hydrothermal strategy that involves coupling low-conductivity NiFe-LDH with 3D porous graphene aerogel (GA) is proposed. The optimized NiFe-LDH/GA (1:1) produced possesses a 257 mV (10 mA cm^−2^) overpotential and could operate stably for 56 h in an OER. Our investigation demonstrates that the NiFe-LDH/GA has a three-dimensional mesoporous structure, and that there is synergistic interaction between LDH and GA and interfacial reconstruction of NiOOH. Such an interface synergistic coupling effect promotes fast mass transfer and facilitates OER kinetics, and this work offers new insights into designing efficient and stable GA-based electrocatalysts.

## 1. Introduction

The search for sustainable energy sources and technologies to solve the energy and ecological problems stemming from the consumption of limited fossil fuels has emerged as a critical priority [1]. Hydrogen production through water splitting is a promising means by which to obtain high purity hydrogen [2,3], and one which cannot only directly use the electricity generated by renewable energy but can also provide a new way to solve the problem of the intermittency of renewable energy [4,5]. However, due to the adverse kinetic and energy barriers of the anodic oxygen evolution reaction (OER), high energy consumption and low efficiency are inevitable. Therefore, OER is the key factor that restricts the whole process [6]. It is vital and urgent to explore advanced OER catalysts with high catalytic activity and high stability.

Transition metals (TMs) have the advantages of being abundant, environmentally friendly and inexpensive, so researchers have made a lot of studies on effective electrocatalysts of TMs, including alloys [7], LDHs [8], transition metal oxides [9], perovskites [10] and sulfur group compounds [11]. In particular, LDHs have been widely used in the fields of electrolysis and electrolytic catalysis, which is attributed to their affordability, high theoretical activity, diverse composition and simple preparation [12,13,14]. However, LDHs have insufficient electrical conductivity and are prone to problems such as build-up and aggregation [15]. This seriously affects the exposure of the more active center of the catalyst and the electron transfer process, thus, greatly affecting its catalytic activity and stability [16].

Various schemes have been proposed to improve their conductivity and stability, with a very feasible approach being the integration of LDHs with conductive carbon substances, like carbon nanotube (CNT), graphene oxide (GO) [17,18], MXenes and nitrogen doped carbon [19,20]. The resulting catalysts can improve the drawbacks of LDHs, thereby substantially enhancing the OER’s performance. For example, Wang et al. reported that nanodots of NiFe-LDH with a high concentration (47 wt%) were cultivated on N-doped 3D porous carbon substances. The NiFe-LDH/3D MPC’s distinctive architecture addresses NiFe-LDH’s issues with lower conductivity and less exposure to active sites, leading to outstanding ORR and OER characteristics [21]. Rinawati et al. synthesized novel doped heterogeneous atoms (GQDs) and doped them into MOF-derived NiFe-LDH. The catalyst exhibits very high catalytic activity in alkaline media [22]. Amin et al. reported that the OER and ORR performances are greatly improved by constructing bimetallic catalysts, and with the assistance of their synergistic effects [23,24]. Although LDH composites have made good progress in electrocatalytic hydrogen and oxygen production, the two-dimensional conductive substrate is not a good solution to the problems of easy aggregation of LDHs and slow mass transfer rate. Therefore, it is necessary to construct LDH composites with 3D conductivity substrate. GA has the benefits of a large amount of porosity, superior electrical conductivity, resistance to corrosion, extensive specific surface area, fast mass/charge transport and stable electrocatalytic performance, and is considered an ideal conductive substrate [25,26,27].

Based on the above considerations, a composite catalyst combining NiFe-LDHs with 3D porous GA was prepared through a simple hydrothermal reaction and the following freeze-drying process. The unique 3D porous structure, large specific surface area and the interfacial synergistic coupling effect between GA and metal active sites jointly improved the charge transfer process and reaction kinetics of the OER. The results show that NiFe-LDH/GA (1:1) exhibits a low overpotential of ~257 mV (10 mA cm^−2^), and that the Tafel slope is down to 49 mV dec^−1^, and displays excellent electrochemical stability for over 56 h of continuous electrocatalysis in the OER. In conclusion, this endeavor provides a viable approach for the development of cost-effective aerogel composites for potential catalysis, energy storage and energy applications.

## 2. Experimental Section

### 2.1. Synthesis of NiFe-LDH

In a typical synthesis of NiFe-LDH (2:1), 1.34 mmol of Ni(NO_3_)_2_·6H_2_O, 0.66 mmol of Fe(NO_3_)_3_·9H_2_O and 1 mmol of urea were dissolved in a mixed solution of deionized water (DIW, 2 mL) and ethylene glycol (EG, 8 mL). The blend was moved to a Teflon-lined steel autoclave following 30 min of agitation. Then, the autoclave was heated at 180 ℃ for 12 h. After cooling down, the sample was washed several times, then dried by freeze-dryer. The synthesis process of NiFe-LDH (3:1), NiFe-LDH (1:1), NiFe-LDH (1:2) and NiFe-LDH (1:3) is the same as that of NiFe-LDH (2:1), with the only difference being the molar ratio of nickel and iron metal salts, respectively.

### 2.2. Synthesis of 3D NiFe-LDH/GA

Three-dimensional NiFe-LDH/GA was prepared by adding different qualities of NiFe LDH to 10 mL of GO solution (2 mg·mL^−1^) and sonicate for 3 h. Then, the mixture was transferred to a Teflon-lined steel autoclave and heated at 180 °C for 6 h. After cooling, the samples were subjected to freeze-drying. The obtained material was named as NiFe-LDH/GA. Different catalysts were synthesized using the same method, except for our adjusting the mass ratio of NiFe-LDH to GO to 2:1, 1:1 and 1:2.

### 2.3. Characterization

An X-ray diffractometer (Bruker D8 ADVANCE*, Bremen, Germany) was used to characterize the crystal structures of the different NiFe-LDH/GA samples. A scanning electron microscopy (SEM, SU-8010, Hitachi, Tokyo, Japan) and a field-emission transmission electron microscope (FEI, Talos-200, Hillsboro, OR, USA) were used to characterize the morphology of the samples. The elemental components and chemical states of samples were measured by X-ray photoelectron spectroscopy (XPS, PHI5000 VersaprobeIII XPS, ULVAC-PHI, Tokyo, Japan). The elemental distribution of NiFe-LDH/GA was determined by EDS mapping at an accelerating voltage of 200 kV.

### 2.4. Electrochemical Measurements

All electrochemical tests of the OER were recorded with an electrochemical workstation (CHI660E, CH Instrument Ins, Shanghai, China) using a three-electrode system, with Hg/HgO (1 M KOH) used as the reference electrodes, while Pt foil and CP loaded with catalyst (1 × 0.5 cm^−2^) were employed as counter and working electrodes, respectively. The working electrode was prepared by the following method: 5 mg of sample, 50 μL of Nafion (5 wt.%) and 950 μL of ethanol solution were mixed by sonication. Then, 30 μL of the inks obtained in the previous step was added to CP (1 × 0.5 cm^−2^), and subjected to natural drying. The amount of material load on the CP was about 0.3 mg cm^−2^. Commercial catalyst (RuO_2_) was used as a comparison. Performance of the OER was evaluated using cyclic voltammetry (CV) and linear sweep voltammetry (LSV) (with scanning speeds of 50 mV s^−1^ and 5 mV s^−1^, respectively). Electrochemical impedance spectroscopy (EIS) was performed in 1 M KOH solution over a frequency range of 0.01 Hz–100 kHz, with an amplitude of 5 mV. Electrochemical double-layer capacitances (C_dl_) were measured via cyclic voltammograms from 1.124 to 1.224 V vs. RHE, where Faradic current was absent. C_dl’_s value was determined by plotting half the variance between anodic and cathodic current densities in relation to the scanning rate. The catalyst was subjected to long-term cycling testing at a constant current density (10 mA cm^−2^). All the measured potentials were standardized to the reversible hydrogen electrode (RHE) using the equation: *E*_RHE_ = 0.0977 V + 0.059 × pH + *E*_Hg/HgO_.

## 3. Results and Discussion

**Figure 1:**
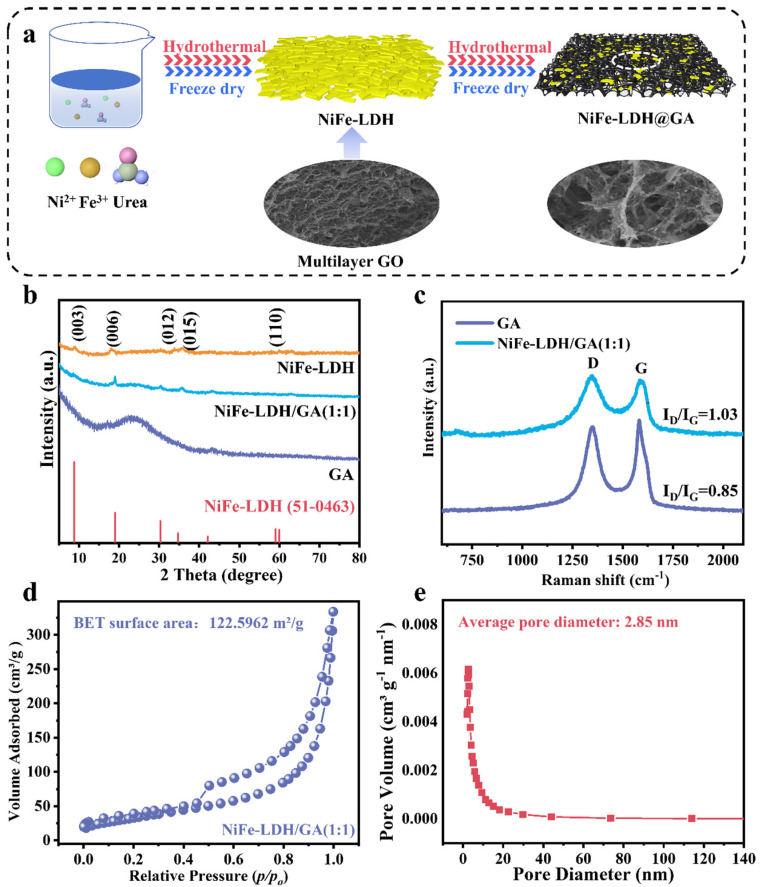
(**a**) Schematic diagram of the synthesis procedure of NiFe-LDH/GA. (**b**) XRD patterns. (**c**) Raman spectra. (**d**) N_2_ adsorption-desorption isotherms. (**e**) Pore size distributions of the NiFe-LDH/GA (1:1).

At first, the 3D NiFe-LDH/GA catalyst was prepared by two-stage hydrothermal reaction and the freeze-drying processes, as depicted in Figure 1a. The physical phase composition and crystal structure of NiFe-LDH/GA and NiFe-LDH were characterized by the X-ray diffraction (XRD) technique (Figure 1b). For NiFe-LDH, the peaks at 9.75°, 18.04°, 33.75°, 36.76° and 60.1° are indexed to (003), (006), (012), (015) and (110) facets of NiFe-LDH (PDF # 51-0463) [28], indicating successful synthesis of NiFe-LDH/GA. However, the peak intensity is faint, indicating the low-crystalline nature. In addition, as the amount of the element Fe increases, this only displays the characteristic peak of FeOOH (Figure S1). When NiFe-LDH was integrated with GA, the NiFe-LDH/GA still has the characteristic peaks of the NiFe-LDH material, indicating that GA does not change the lamellar structure. It is worth noting that the peak strength of the material became higher, indicating that the addition of graphene oxides in the secondary hydrothermal process enhanced the crystallinity of NiFe-LDH, which proves the strong interaction of the two components. Compared with NiFe-LDH/GA (1:1) and NiFe-LDH/GA (2:1), the characteristic peaks of NiFe-LDH/GA (1:2) are weaker, which may be due to less NiFe-LDH being added (Figure S2).

In the Raman spectra (Figure 1c), the intensity ratios (I_D_/I_G_) of the D and G bands of NiFe-LDH/GA (1:1) and GA are 1.03 and 0.85, respectively. This suggests that the integration of NiFe-LDH with GA leads to an increase in the number of defects on the GA surface. From Figure 1d, it can be seen that the adsorption isotherm of NiFe-LDH/GA (1:1) is similar to the characteristics of the type IV adsorption isotherm, and presents an obvious h3-type hysteresis line, which suggests that the material has a mesoporous structure [29]. The specific surface area of NiFe-LDH/GA (1:1) is measured to be 122.59 m^2^/g, which is significantly higher than that of the previously documented LDH/GA composites [30,31]. Meanwhile, Figure 1e indicates that the average pore diameter of the composites is around 2.85 nm, further confirming the mesoporous structure of NiFe-LDH/GA (1:1). From Figure S11, it can be seen that the specific surface area of the GA is 153.43 m^2^/g and that of NiFe-LDH is 10.56 m^2^/g. Compared to the GA, the specific surface area of NiFe-LDH/GA (1:1) decreases, which may be attributed to the fact that the composite of NiFe-LDH causes some of the pores of the GA to be blocked. The larger specific surface area provides more active sites, and the mesoporous structure is conducive to reactant transportation and immediate desorption of gases, which, in turn, improves the catalytic activity.

**Figure 2:**
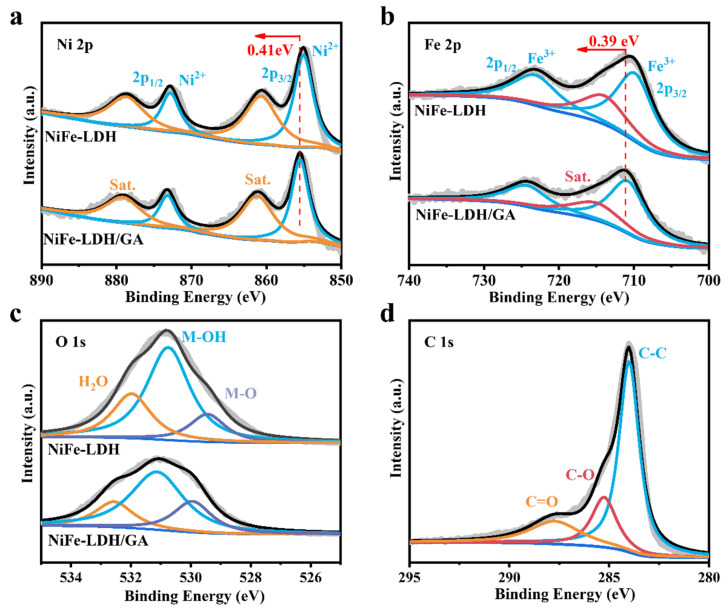
(**a**) Ni 2p, (**b**) Fe 2p and (**c**) O 1s XPS of NiFe-LDH/GA and NiFe-LDH. (**d**) C 1s XPS of NiFe-LDH/GA.

The elemental composition and valence states of the catalysts were subsequently characterized using XPS. The overall spectrum of NiFe-LDH/GA (1:1) and NiFe-LDH (Figure S3) manifested the coexistence of Ni, Fe and O. Figure 2a shows the Ni 2p spectra of NiFe-LDH, where the Ni^2+^, 2p_3/2_ and 2p_1/2_ located at 855.12 and 872.88 eV and accompanied by two satellite peaks located at 860.78 and 878.78 eV, respectively, can be seen [31,32]. The Fe 2p spectrum of NiFe-LDH in Figure 2b shows Fe^3+^, 2p_3/2_ and 2p_1/2_ located at 709.85 and 723.11 eV, which are quintessential Fe^3+^ peaks, demonstrating that Fe exists in the form of Fe^3+^ in NiFe-LDH/GA (1:1), and that there is also a satellite peak (noted as Sat.) located at 713.93 eV [32,33]. Notably, compared with NiFe-LDH, the Ni and the Fe 2p peak of NiFe-LDH/GA (1:1) were shifted positively by 0.41 and 0.39 eV, respectively, demonstrating that there is a strong electronic interaction and electronic structure change on the interface of NiFe-LDH and GA [32,34]. Figure 2c illustrates the distinct peaks of O 1s of NiFe-LDH/GA (1:1), which can be deconvoluted into three characteristic peaks located at 530.2, 531.2 and 532.63 eV, which are assigned to M-O, M-OH and adsorbed water on the surface [35], respectively. The C 1s spectra of NiFe-LDH/GA (1:1) (Figure 2d) show that the three characteristic peaks at 284.09, 285.34 and 287.81 eV were assigned to the bonds of C-C, C-O and C=O [36], respectively. The intensity of the C 1s peak was reduced compared to that of the pre-OER test. However, there still exist three chemical bonds C-C, C-O and C=O (Figure S10). The above results further demonstrate the strong electronic interaction between NiFe-LDH and GA. This strong electronic interaction helps to enhance the catalytic activity and stability of the catalyst [36].

**Figure 3:**
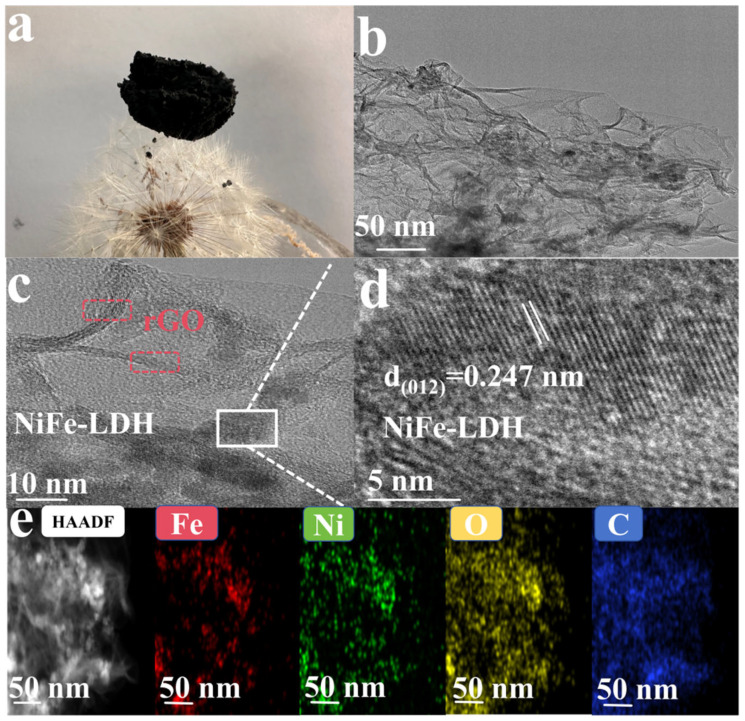
(**a**) Optical photographs of NiFe-LDH/GA (1:1); (**b**) TEM image; (**c**,**d**) HRTEM images of NiFe-LDH/GA (1:1) and (**e**) EDX elemental mapping images of NiFe-LDH/GA (1:1).

Figure 3a is a photograph of the NiFe-LDH/GA (1:1) composite aerogel. When the whole NiFe-LDH/GA (1:1) composite aerogel was placed on the dandelion, there was no deformation of the dandelion, which indicates that the NiFe-LDH/GA (1:1) composite has an ultra-low density. The NiFe-LDH microstructure was found to be a nanoflower composed of nanosheets (Figure S4). The stacking of these nanoflowers leads to under-exposure of active sites, which, in turn, reduces the catalytic activity. The original GA is a typical multilayer structure (Figure S5). It was observed from Figure S6 that the microstructure of NiFe-LDH/GA (1:1) is composed of interwoven and entangled rGO nanoflakes, which form a rich 3D pore structure. This unique 3D structure has a large specific surface area conducive to the loading of NiFe-LDH and to preventing the aggregation of NiFe-LDH, allowing unhindered diffusion and penetration of electrocatalytic active species and, thus, improving the catalytic activity. From the scanning electron microscope images (Figure S6), it can be seen that the microscopic morphology of the different samples still maintains the three-dimensional network structure. However, when the amount of NiFe-LDH was larger than that of graphene oxide, there was an obvious stacking phenomenon between the graphene flakes, which led to an obvious reduction in the number of pores in the NiFe-LDH/GA (2:1), which, in turn, might lead to a decrease in the catalytic activity. Transmission electron microscope (TEM) images (Figure 3b,c) show that NiFe-LDH is evenly distributed across the surface of ultra-thin rGO nanosheets that are full of folds [37]. In the corresponding HRTEM images, NiFe-LDH (012) lattices with a spacing of 0.247 nm can be observed (Figure 3d) [38], and the corresponding graphite lattices of graphene flakes are also clearly visible. Subsequently, the HAADF-STEM and its corresponding EDX elemental mapping (Figure 3e) revealed a homogeneous distribution of the elements O, Ni and Fe in FeNi-LDH/GA (1:1).

**Figure 4:**
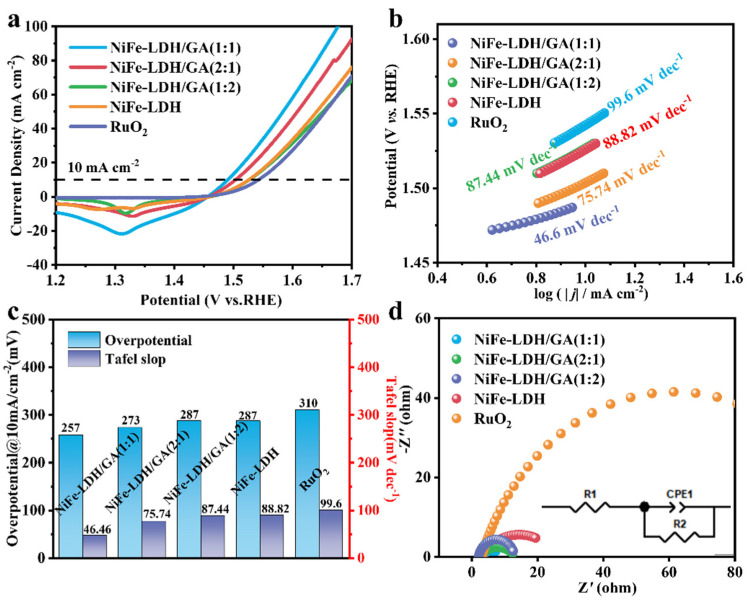
(**a**) CV curves, (**b**) Tafel plots analysis, (**c**) histogram of Tafel values and overpotentials at 10 mA cm^−2^ and (**d**) The EIS Nyquist plots of NiFe-LDH, NiFe-LDH/GA (1:1), NiFe-LDH/GA (1:2), NiFe-LDH/GA (2:1) and commercial RuO_2_ catalysts.

The OER performance of different samples in a 1 M KOH solution was evaluated using a three-electrode measurement system. Firstly, the effect of the ratio of metal salts on the catalytic activity of NiFe-LDH was explored (Figure S7). NiFe-LDH has the maximum ECSA when the Ni:Fe ratio is 2:1, which indicates that it has more active sites, which gives optimal catalytic activity. Subsequently, the impact of the mass ratio of NiFe-LDH to GO on the catalytic performance of NiFe-LDH/GA composites was examined (Figure 4a). The overpotential of NiFe-LDH/GA (1:1) was 257 mV at 10 mA cm^−2^, which is lower than that of NiFe-LDH/GA (2:1) (273 mV), NiFe-LDH/GA (1:2) (287 mV), RuO_2_ (310 mV) and NiFe-LDH (287 mV). Typically, the reaction kinetics of a catalyst are evaluated based on the Tafel slope. As illustrated in Figure 4b,c, the Tafel slope for NiFe-LDH/GA (1:1) was only 46.46 mV dec^−1^, which was much lower than that for NiFe-LDH (1:2) (87.44 mV dec^−1^), NiFe-LDH (2:1) (75.74 mV dec^−1^), NiFe-LDH (88.82 mV dec^−1^) and RuO_2_ (99.6 mV dec^−1^). As expected, the NiFe-LDH/GA (1:1) has the best electrocatalytic OER kinetics. In comparison, it can be demonstrated that the integration of NiFe-LDH with GA is conducive to accelerating the kinetics of the OER reaction [39].

Electrochemical impedance spectroscopy (EIS) tests were conducted to look deeper into the reaction rates of various catalysts (Figure 4d). The NiFe-LDH/GA (1:1) has the smallest concave semicircle on the Nyquist plot, and its R_ct_ is only 7.34 Ω, which is smaller than that of NiFe-LDH/GA (2:1) (11.02 Ω), NiFe-LDH/GA (1:2) (11.14 Ω), NiFe-LDH (23.52 Ω) and RuO_2_ (117.85 Ω) (Table S1). Compared with NiFe-LDH, the resistance of the composite material with GA is significantly reduced. This may be due to the fact that the excellent conductivity of GA improves the conductivity of NiFe-LDH, which, in turn, leads to a significant reduction in the resistance of the composite material. Therefore, NiFe-LDH/GA (1:1) has faster reaction kinetics and a faster charge transfer rate during the OER procedure. It is shown that integrating NiFe-LDH with GA not only effectively accelerates the reaction kinetics, but also enhances the electrical conductivity, which leads to the enhancement of OER activity. Meanwhile, the catalytic activity of NiFe-LDH/GA (1:1) was superior to that of most reported LDHs materials and other non-precious metal electrocatalytic catalysts (Table S2). In addition, employing C_dl_ measurements allowed us to calculate the ECSA directly, thus, further investigating the impact on catalytic performance [40]. The C_dl_ value of the NiFe-LDH/GA (1:1) catalyst (13.41 mF·cm^−2^) was higher than that of the NiFe-LDH catalyst (1.05 mF·cm^−2^) (Figure S8). The ECSA is directly proportional to the C_dl_. The larger ECSA implies that the catalyst has more active centers and higher catalytic activity. This indicates that the synergistic interface coupling of NiFe-LDH and GA can result in an increase in the active area and, thereby, can contribute to the enhanced catalytic activity in the OER [41,42].

**Figure 5:**
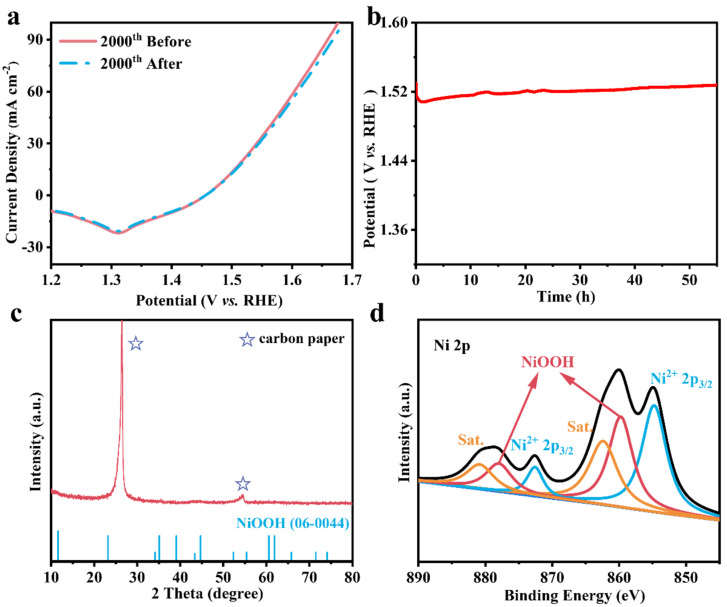
(**a**) CV curves before and after 2000 CV cycles. (**b**) Long-term durability tests conducted at 10 mA·cm^−2^. (**c**) XRD of NiFe-LDH/GA (1:1) after OER. (**d**) Ni 2p of NiFe-LDH/GA after OER.

The durability under an alkaline environment is an important consideration when evaluating catalyst performance. Therefore, we performed stability tests on NiFe-LDH/GA (1:1). The CV did not show a notable reduction following 2000 successive cycles (Figure 5a). After 56 h of long-term stability testing, only a 20 mV increase in overpotential was detected, indicating that the catalyst showed excellent durability and efficiency (Figure 5b). The SEM images in Figure S9 show that the similar layered structure of LDH and the three-dimensional skeleton of graphene are well preserved during operation, which also reveals the effectiveness of the synergistic effect of NiFe-LDH with GA. The post-test XRD analysis showed the extremely feeble peaks at 35°–45°, corresponding to NiOOH (PDF # 06-0044) [43], which is due to the fact that the NiOOH is produced by the auto-oxidation process from Ni^2+^ to Ni^3+^ during the OER process, and is only distributed on the surface and remains in an amorphous state (Figure 5c) [44]. The elemental composition and valence of NiFe-LDH/GA (1:1) after the long cycle test were analyzed by XPS. As shown in Figure S10, after 56 h of water oxidation, there were no significant changes in Fe 2p and O 1s. As for the Ni 2p high-resolution XPS, two new peaks appeared at 859.76 and 877.97 eV (Figure 5d), which corresponded to the formation of NiOOH after the OER [44]. This phenomenon also indicates that catalyst reconstruction occurred on the surface of the GA during the OER, and that the newly formed NiOOH directly acted as the real active site [45]. The above test results show that the NiFe-LDH/GA (1:1) catalyst has good catalytic durability.

## 4. Conclusions

In conclusion, an efficient and stable OER electrocatalyst (NiFe-LDH/GA) was successfully prepared. This profited from excellent electrical conductivity, large specific surface area, numerous active sites and three-dimensional porous channels. The optimized NiFe-LDH/GA (1:1) catalyst possessed a 257 mV (10 mA cm^−2^) overpotential and good OER stability over 56 h in 1 M KOH, which are superior to most reported NiFe-LDHs and/or commercial RuO_2_ catalysts. The synthesis proposed in this work is also applicable to other types of LDH/GA composites. It offers a new approach for the synthesis of efficient, stable GA-based composites with finely-modulated porous structures and satisfies the requirements of catalytic performances.

## Data Availability

Data is contained within the article.

## Supplementary Materials

The following supporting information can be downloaded at: https://www.mdpi.com/article/10.3390/nano14201661/s1, Figure S1. XRD of NiFe-LDH prepared by varying the ratio of metal salts. Figure S2. XRD of NiFe-LDH/GA and NiFe-LDH. Figure S3. XPS survey spectra of NiFe-LDH/GA (1:1) and NiFe-LDH. Figure S4. SEM images of NiFe-LDH (2:1). Figure S5. SEM images of GA. Figure S6. SEM images of (a,b) NiFe-LDH/GA (1:1); (c,d) NiFe-LDH/GA (1:2); (e,f) NiFe-LDH/GA (2:1). Figure S7. CV of samples with different metal ratios. Figure S8. The ECSA of (a) NiFe-LDH, (b) NiFe-LDH/GA (1:2), (c) NiFe-LDH (1:1)/GA, (d) NiFe-LDH/GA (2:1), (e) RuO_2_ at different sweep speeds, (f) Cdl of different samples in 1 M KOH. Figure S9. SEM for NiFe-LDH/GA (1:1) after 56 h of long cycle testing. Figure S10. XPS for NiFe-LDH/GA (1:1) after 56 h of long cycle testing. (a) O1s; (b) Fe 2p, (c) C 1 s. Figure S11. N_2_ adsorption-adsorption isotherms for (a) GA; (b) NiFe-LDH. Figure S12. The ECSA of (a) NiFe-LDH (3:1), (b) NiFe-LDH (2:1), (c) NiFe-LDH (1:1). Table S1. Impedance parameter values obtained by fitting the Nyquist curve of the equivalent circuit OER. Table S2. Comparison of the OER performance of various related electrocatalysts at a current density of 10 mA cm^−2^. Refs. [14,37,46,47,48,49,50,51,52,53,54,55] are cited in the Supplementary Materials.
