# Supplementary material for: Interface Synergistic Effect of NiFe-LDH/3D GA Composites on Efficient Electrocatalytic Water Oxidation
Source: Nanomaterials (Basel). 2024 Oct 16;14(20):1661. doi: 10.3390/nano14201661 (PMC11510525; doi:10.3390/nano14201661)
Supplement: Supplementary file 1 [file nanomaterials-14-01661-s001.zip › nanomaterials-3259104-supplementary.pdf]

Supplementary File

Interface Synergistic Effect of NiFe-LDH @ 3D GA Composites towards Efficient Electrocatalytic Water Oxidation

Jiangcheng Zhang[a], Qiuhan Cao[a], Xin Yu[a], Hu Yao[a], Baolian Su[b], Xiaohui Guo[a,*]

[a]Key Lab of Synthetic and Natural Functional Molecule Chemistry of Ministry of Education, The College of Chemistry and Materials Science, Northwest University, Xi'an 710069, P. R. China.

[b]Department of inorganic chemistry, University of Namur, 61 rue de Bruxelles, B-5000 Namur, Belgium.

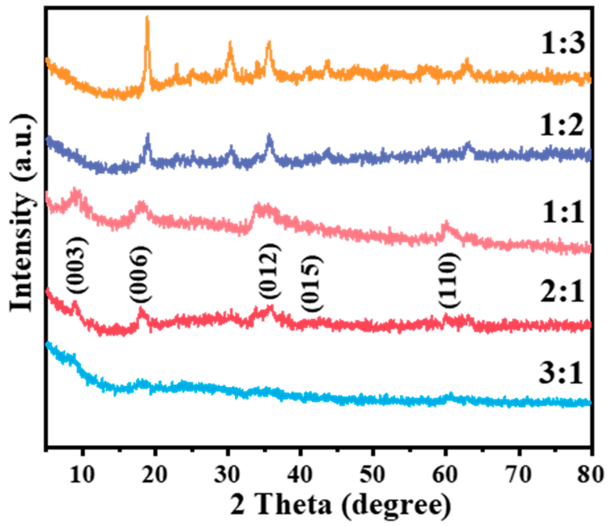

Figure S1. XRD of NiFe-LDH prepared by varying the ratio of metal salts.

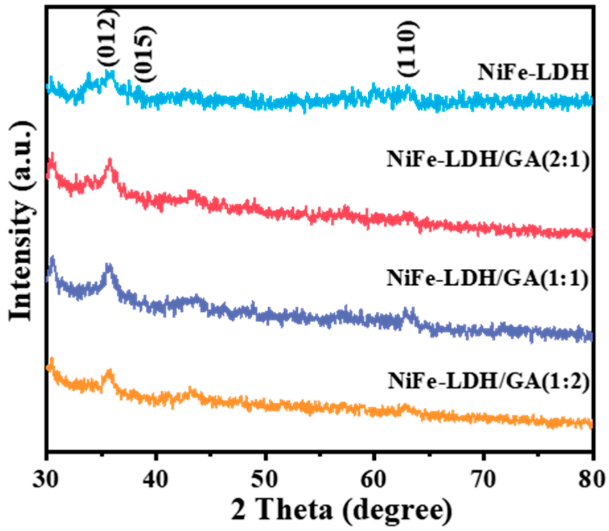

Figure S2. XRD of NiFe-LDH/GA and NiFe-LDH.

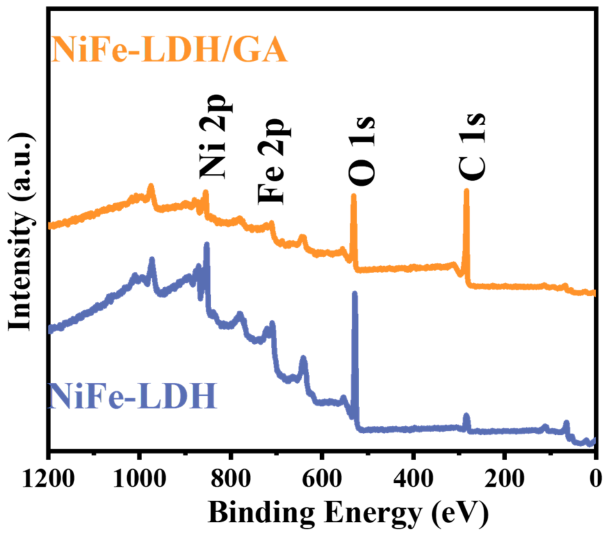

Figure S3. XPS survey spectra of NiFe-LDH/GA (1:1) and NiFe-LDH.

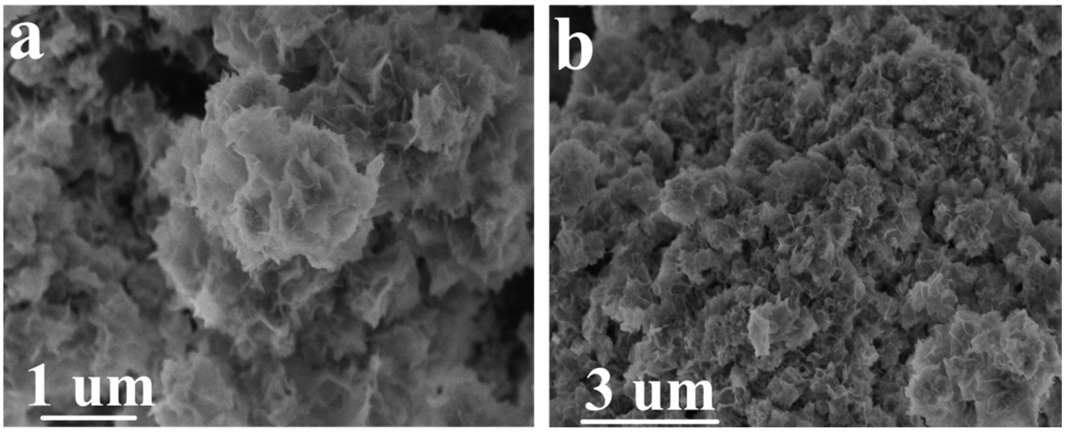

Figure S4. SEM images of NiFe-LDH(2:1).

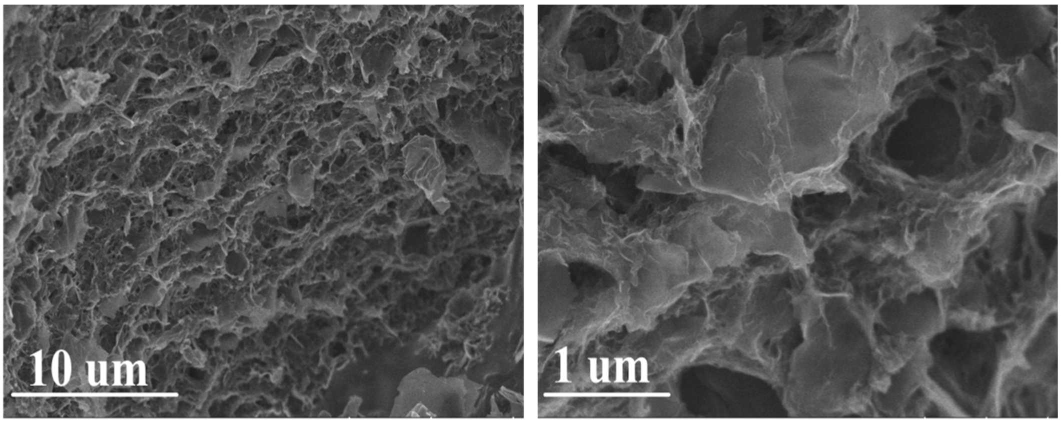

Figure S5. SEM images of GA.

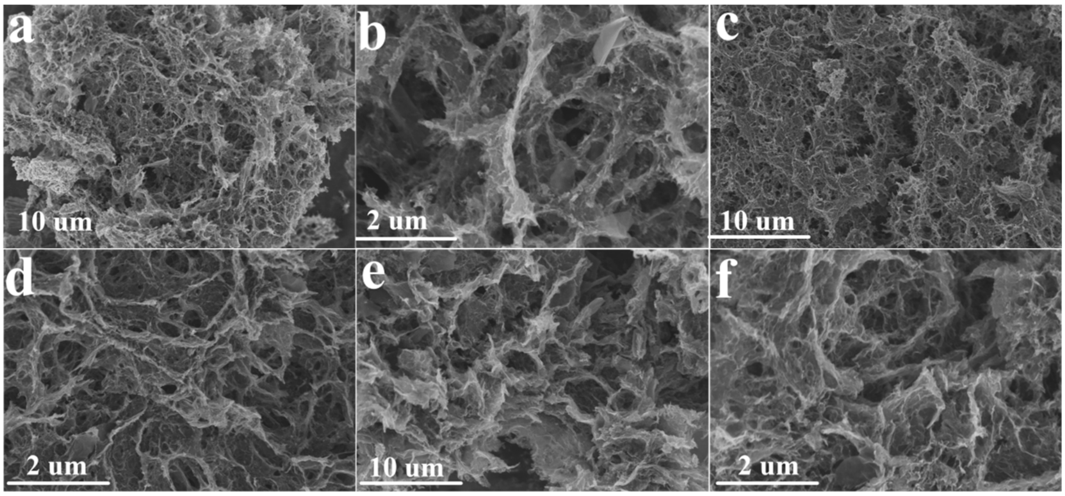

Figure S6. SEM images of (a-b) NiFe-LDH/GA (1:1); (c-d) NiFe-LDH/GA(1:2); (e-f) NiFe-LDH/GA (2:1)

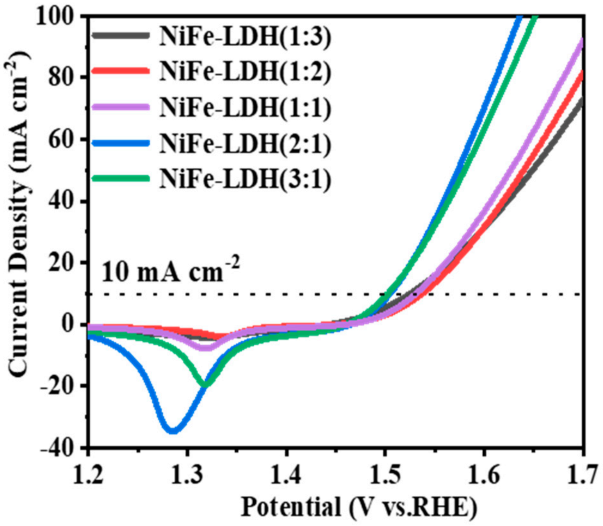

Figure S7. CV of samples with different metal ratios.

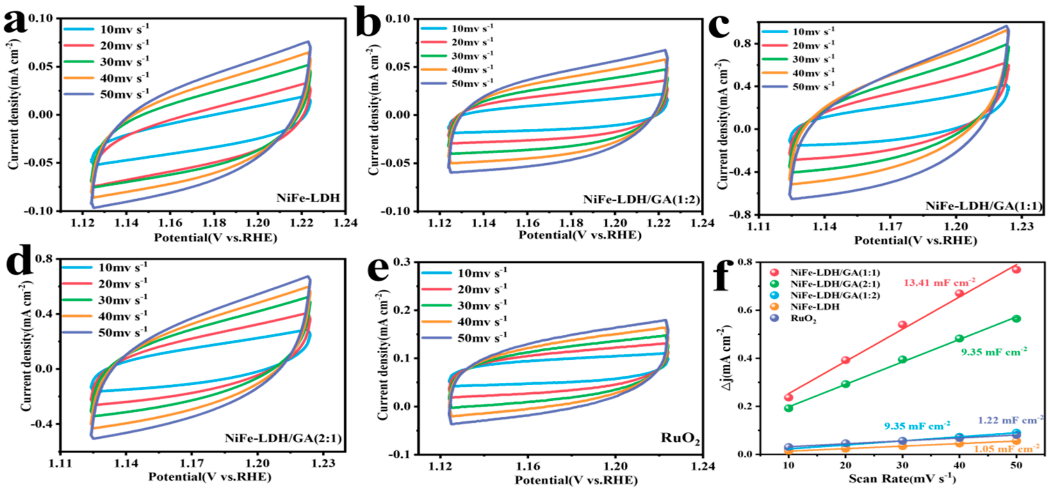

Figure S8. The ECSA of (a)NiFe-LDH, (b) NiFe-LDH/GA(1:2), (c) NiFe-LDH(1:1)/GA, (d) NiFe-LDH/GA(2:1), (e) RuO_2 at different sweep speeds, (f) C_{dl} of different samples in 1 M KOH.

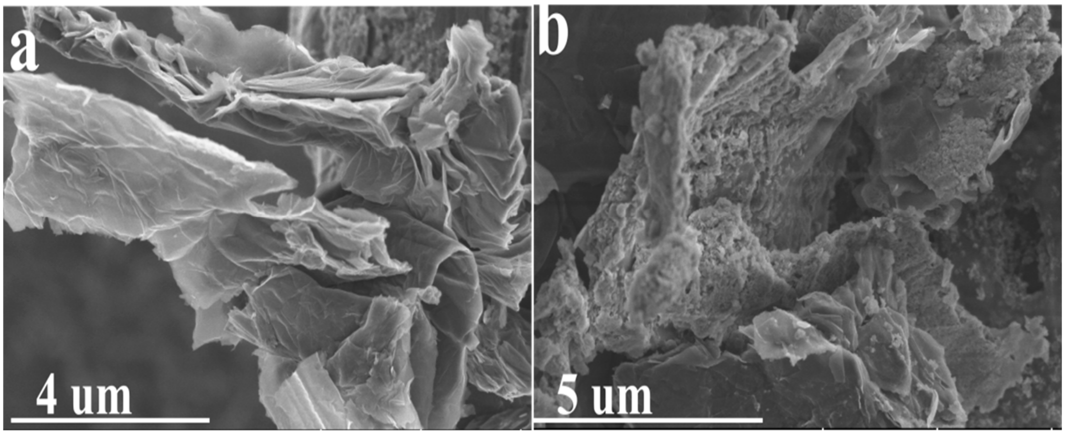

Figure S9. SEM for NiFe-LDH/GA(1:1) after 56h of long cycle testing.

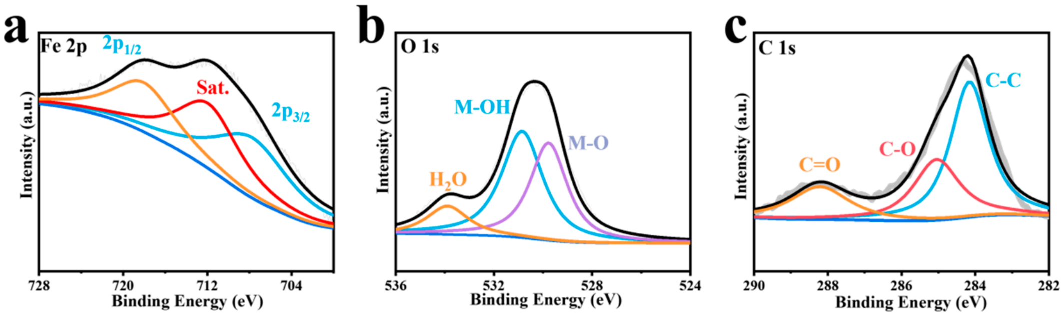

Figure S10. XPS for NiFe-LDH/GA(1:1) after 56h of long cycle testing. (a) O1s; (b) Fe 2p, (c) C 1s.

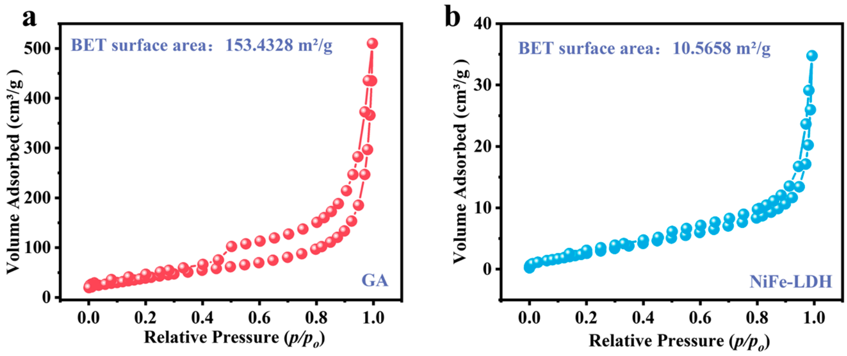

Figure S11. N_2 adsorption-adsorption isotherms for (a) GA; (b)NiFe-LDH.

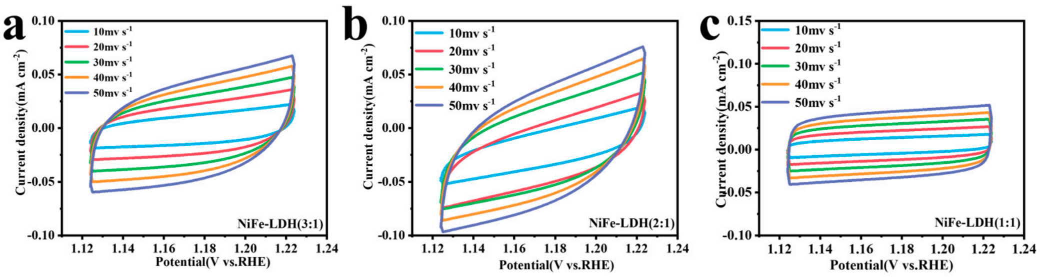

Figure S12. The ECSA of (a)NiFe-LDH(3:1), (b) NiFe-LDH(2:1), (c) NiFe-LDH(1:1).

Table S1. Impedance parameter values obtained by fitting the Nyquist curve of the equivalent circuit OER

Samples	R_1	R_s	R_{ct}
NiFe-LDH/GA(1:1)	0.504	2.509	7.341
NiFe-LDH/GA(1:2)	0.564	2.736	11.14
NiFe-LDH/GA(2:1)	0.53	2.352	11.02
NiFe-LDH	0.851	2.063	23.52
RuO_2	0.787	2.862	117.85

Table S2. Comparison of the OER performance of various related electrocatalysts at a current density of 10 mA cm^{-2}.

Catalysts	η10 mA cm^{-2} (mV)	Tafel slope (mV dec^{-1})	Reference
NiFe-LDH/GA(1:1)	257	46.46	This work
NiFeCo-LDH/CF	249	42	1
ReS_2/NiFe-LDH	266	43	2
CoCr LDH	338	74	3
FeCoNi-LDHs	269	42.34	4
La-NiFe LDH	340	97.1	5
NiCo LDH-TPA	267	52.4	6
NiCo-LDH@MOFs	289	55.2	7
NiFeRh-LDH	204	29	8
CeO_2-FeCo LDH	280	42	9
NiCo-LDH/GO-CNTs	290	66.8	10
Ni-Fe LDH	280	49.4	11
NiFe-LDH/CNT@GNR	261	78	12

References

1. Lin, Y. P.; Wang, H.; Peng, C. K.; Bu, L. M.; Chiang, C. L.; Tian, K.; Zhao, Y.; Zhao, J. Q.; Lin, Y. G.; Lee, J. M.; Gao, L. J., Co-Induced Electronic Optimization of Hierarchical NiFe LDH for Oxygen Evolution. *Small* **2020,** *16* (38), 2002426.

2. Han, X. T.; Li, N. N.; Kang, Y. B.; Dou, Q. Y.; Xiong, P. X.; Liu, Q.; Lee, J. Y.; Dai, L. M.; Park, H. S., Unveiling Trifunctional Active Sites of a Heteronanosheet Electrocatalyst for Integrated Cascade Battery/Electrolyzer Systems. *ACS Energy Lett.* **2021,** *6* (7), 2460-2468.

3. Meng, H. Y.; Xi, W.; Ren, Z. Y.; Du, S. C.; Wu, J.; Zhao, L.; Liu, B. W.; Fu, H. G., Solar-boosted electrocatalytic oxygen evolution via catalytic site remodelling of CoCr layered double hydroxide. *Appl. Catal., B* **2021,** *284*, 119707.

4. Hu, Y. D.; Luo, G.; Wang, L. G.; Liu, X. K.; Qu, Y. T.; Zhou, Y. S.; Zhou, F. Y.; Li, Z. J.; Li, Y. F.; Yao, T.; Xiong, C.; Yang, B.; Yu, Z. Q.; Wu, Y., Single Ru Atoms Stabilized by Hybrid Amorphous/Crystalline FeCoNi Layered Double Hydroxide for Ultraefficient Oxygen Evolution. *Adv. Energy Mater.* **2021,** *11* (1), 2002816.

5. Yu, J.; Lu, K.; Wang, C. X.; Wang, Z. M.; Fan, C. C.; Bai, G.; Wang, G.; Yu, F., Modification of NiFe layered double hydroxide by lanthanum doping for boosting water splitting. *Electrochim Acta* **2021,** *390*, 138824.

6. Liu, W. X.; Zheng, D.; Deng, T. Q.; Chen, Q. L.; Zhu, C. Z.; Pei, C. J.; Li, H.; Wu, F. F.; Shi, W. H.; Yang, S. W.; Zhu, Y. H.; Cao, X. H., Boosting Electrocatalytic Activity of 3d-Block Metal (Hydro)oxides by Ligand-Induced Conversion. *Angew Chem Int Edit* **2021,** *60* (19), 10614-10619.

7. Han, M. Y.; Zhang, X. W.; Gao, H. Y.; Chen, S. Y.; Cheng, P.; Wang, P.; Zhao, Z. Y.; Dang, R.; Wang, G., In situ semi-sacrificial template-assisted growth of ultrathin metal-organic framework nanosheets for electrocatalytic oxygen evolution. *Chem. Eng. J.* **2021,** *426*, 131348.

8. Sun, H. C. A.; Zhang, W.; Li, J. G.; Li, Z. S.; Ao, X.; Xue, K. H.; Ostrikov, K. K.; Tang, J.; Wang, C. D., Rh-engineered ultrathin NiFe-LDH nanosheets enable highly-efficient overall water splitting and urea electrolysis. *Appl. Catal., B* **2021,** *284*, 119740.

9. Li, Y. Y.; Luo, W.; Wu, D. J.; Wang, Q.; Yin, J.; Xi, P. X.; Qu, Y. Q.; Gu, M.; Zhang, X. Y.;

Lu, Z. G.; Zheng, Z. P., Atomic-level correlation between the electrochemical performance of an oxygen-evolving catalyst and the effects of CeO functionalization. *Nano Research* **2022,** *15* (4), 2994-3000.

10. Yin, P. Q.; Wu, G.; Wang, X. Q.; Liu, S. J.; Zhou, F. Y.; Dai, L.; Wang, X.; Yang, B.; Yu, H. Q., NiCo-LDH nanosheets strongly coupled with GO-CNTs as a hybrid electrocatalyst for oxygen evolution reaction. *Nano Research* **2021,** *14* (12), 4783-4788.

11. Yu, L.; Yang, J. F.; Guan, B. Y.; Lu, Y.; Lou, X. W., Hierarchical Hollow Nanoprisms Based on Ultrathin Ni-Fe Layered Double Hydroxide Nanosheets with Enhanced Electrocatalytic Activity towards Oxygen Evolution. *Angew Chem Int Edit* **2018,** *57* (1), 172-176.

12. Yin, X.; Hua, Y. N.; Hao, W. B.; Yang, J.; Gao, Z., Hierarchical nanocomposites of nickel/iron-layered double hydroxide ultrathin nanosheets strong-coupled with nanocarbon networks for enhanced oxygen evolution reaction. *Electrochim Acta* **2022,** *420*, 140455.
